# Flexible use of multimodal communicative strategies in adult chimpanzees

**DOI:** 10.1038/s41598-025-14835-x

**Published:** 2025-09-09

**Authors:** Angèle Lombrey, Adriana Luna Martinez, Nick Dannenmann, Katerina Harvati, Ulrich Knief, Marlen Fröhlich

**Affiliations:** 1 Paleoanthropology Section, Department of Geosciences, Institute for Archaeological Sciences, University of Tübingen, Tübingen, Germany; 2 Development and Evolution of Cognition Research Group, Max Planck Institute of Animal Behavior, Konstanz, Germany; 3 DFG Centre for Advanced Studies ‘Words, Bones, Genes, Tools’, University of Tübingen, Tübingen, Germany; 4 Evolutionary Biology and Ecology, Institute of Biology I (Zoology), University of Freiburg, Freiburg, Germany

**Keywords:** Behavioural reaction norms, Context, Individual variation, Multicomponent signalling, Multisensory signalling, *Pan troglodytes*, Plasticity, Biological anthropology, Animal behaviour

## Abstract

Human communication is remarkable for its flexibility, a trait largely reflected in its multimodal nature and shared to some extent with nonhuman primates. Although individual differences in social behaviour are known to have evolutionary implications, their role in shaping primate communication remains largely unexplored. This study adopts a multimodal framework to partition variation in chimpanzees’ use of multicomponent and multisensory communicative strategies into socio-environmental, between-individual, and within-individual sources. Results showed that research setting and signaller’s sex affected communicative expression. Importantly, we also detected consistent between-individual differences in both strategies, independent of age, sex, or setting. While only multicomponent signal use was predicted by behavioural context at the population level, individuals varied in how they adjusted to context only in their use of multisensory acts. These findings reveal substantial flexibility in chimpanzee communication, highlighting individual-specific patterns and supporting a gradual evolutionary pathway toward the complexity of human multimodal communication.

**Supplementary Information:**

The online version contains supplementary material available at 10.1038/s41598-025-14835-x.

## Introduction

Although the key features of human face-to-face communication have been studied for decades and the range of empirical approaches is becoming ever wider^1,2,^ satisfactory answers concerning the evolutionary building blocks of language are yet to be found^3–5^. As our closest living relatives, great apes are a model of choice to address these questions with comparative research. A critical feature of human communication is its extraordinary plasticity, reflected in the flexible production and interpretation of signals based on the common ground shared by interaction partners^4,6^. This is partly manifested in linguistic politeness and other forms of audience design, through which interactants adjust their signalling to one another in order to reduce uncertainty, build and maintain relationships, and adhere to social norms^7,8^. This flexibility might seem redundant and cognitively demanding, but it actually helps reinforce mutual understanding^9^. Great apes’ communication systems are among the most flexible in the animal kingdom: similarly to humans, they are able to combine multiple signals at the same time and adjust their signal use to the social context, the attention state of the interlocutor and to past social interactions (gorillas (*Gorilla gorilla*)^10^; orang-utans (*Pongo spp.*)^11,12^; chimpanzees (*Pan troglodytes*)^13,14^; bonobos (*Pan paniscus*)^15^. This is also reflected in their capacity to persist and elaborate their signalling when their social goal is not achieved^16,17^ and to flexibly dissociate signals and outcomes: a single (e.g. gestural) signal can be used to achieve several purposes, and multiple signals can lead to the same outcome^12,18–20^.

Language is intrinsically multimodal, that is, communicative signals are simultaneously produced across different articulators (multicomponent) and understood across different sensory modalities (multisensory). Although multimodality serves as a key source of language flexibility^21–23^, one major shortcoming of the comparative study of primate communication is that, for decades, it has been approached in a unimodal way^24,25^. For a long time, researchers have been divided into two major camps: proponents of either the gesture-first or the vocal-first theory of language origin both argue that language evolved from one or the other of these two modalities of communication^26,27^. However, multimodality is essential in language expression and integration, as is evidenced by the McGurk effect. That is, our understanding of speech comes from the simultaneous integration of vocal (speech itself) and visual (in this case, facial) components, and different combinations (e.g. unmatched vocal and facial components) result in different speech understandings^28–30^. Considering the pervasiveness of multimodality in interactive language use (where speech is usually accompanied by facial expressions and gestures^31^, the multimodal production of signals by many non-human species^32^ and their importance in social negotiation^33^ a unimodal approach most likely results in the loss of important information concerning language origins. While many human and non-human multimodal signals are fixed^28,34–36^ (vocalizations, including most speech sounds, cannot be expressed without their complementary facial signal), multimodality in signal combination (e.g. combinatory use of vocal and gestural signals) can be adapted to specific environments and enables highly flexible communication^20,37^. In addition, the unimodal approach of communication hinders reliable comparisons between the different modalities^24^. Thus, to gain a more holistic understanding of communicative interactions and enable better comparisons with human communication, a growing number of studies have focused on the combination of signal modalities^37–39^. However, although multimodal approaches are spreading in studies on animal primate communication, they do not investigate multimodal communicative behaviour in all its diversity: studies often focus either on the co-occurrence of signal types (e.g^.40–42^) or of sensory modalities (e.g.^10,13)^. Within this framework, one key goal of this study is to investigate and compare the flexibility of specific multimodal communicative strategies in chimpanzees through a holistic approach.

**Figure 1:**
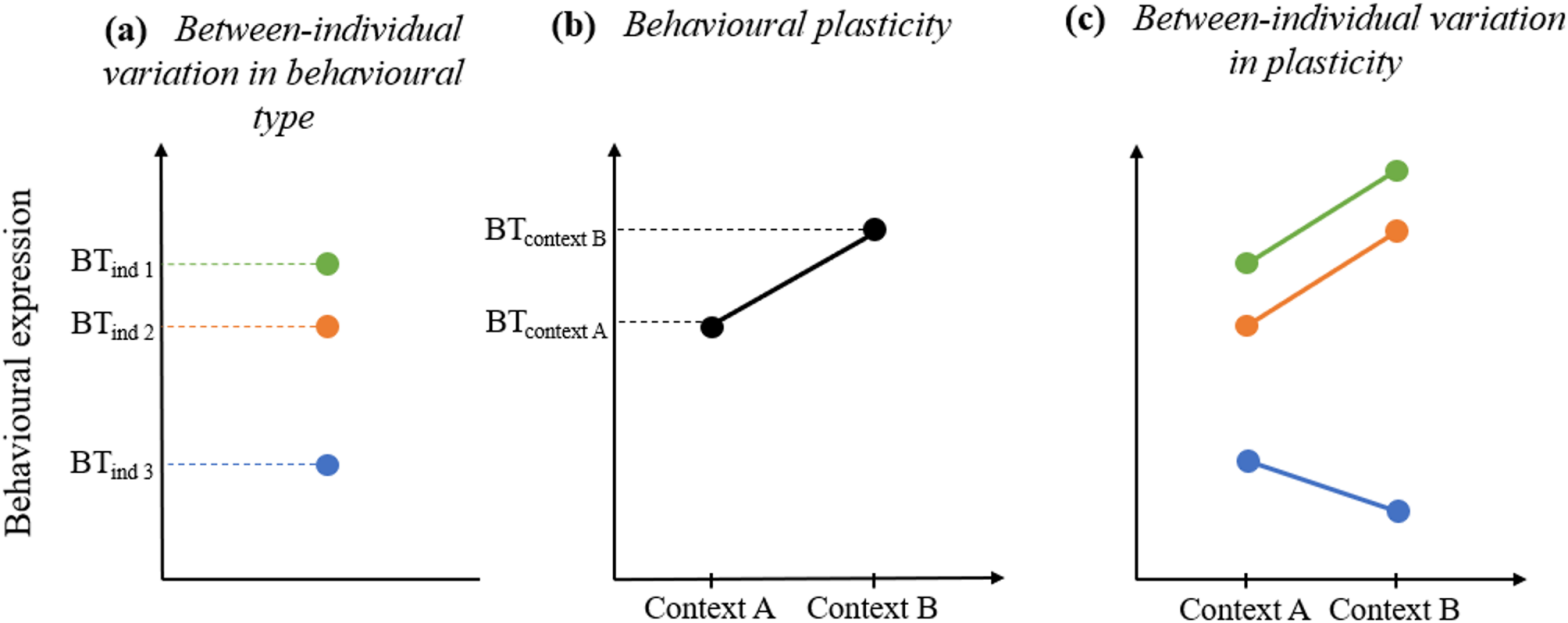
Types of individual variation. In (**a**), individuals differ in their mean behavioural expression (behavioural type, BT). In **(b)**, individuals adjust their behavioural expression across contexts (i.e. they express behavioural plasticity; slope ≠ 0). Individuals with a slope equal to 0 do not express behavioural plasticity. In **(c)**, individuals 1 and 2 exhibit similar plasticity (parallel slopes), while individual 3 differs from them in its plastic response (non-parallel slope). Colours represent different individuals. Adapted from Fröhlich et al.^38^, Hertel et al.^53^ and Dingemanse and Wolf ^52^.

Although multimodal approaches have become more common, another critical problem with current comparative research is that variation in communicative behaviour is mostly studied on the population or species level. These approaches are of great importance to investigate communication systems, but have for consequence the treatment of individual variation as ‘noise’, which is assumed to disappear once sufficient data have been collected. However, between-individual variation in behavioural type, or individual variation in the average expression of behaviours (i.e. personality: *consistent individual differences in social behaviour*, Fig. 1a*)*, has been shown to have biological and ecological importance, whether for population dynamics and survival or for social evolution^43,44,^ including in great ape species^45^. Thus, variation in behavioural and communicative patterns found between populations may often be due to extreme behavioural expression in only a few individuals^38^. Moreover, natural selection acts directly on individual differences, potentially leading to adaptive evolution^46^. Variation in genetics and epigenetics between individuals might not only impact the expression of innate behavioural traits, but also the way these traits are inherited through time in a given population. Additionally, some behavioural traits might be propagated through social learning, with individuals adopting strategies to learn adaptive behaviours from others^47,^ providing a second, social level for selection to act. As a consequence, individual variation is highly biologically relevant and must be taken into account in studies of human behavioural evolution^48^.

Importantly, individuals may not only differ consistently in their average expression of behaviour, but they may also modify their behaviour when the social or environmental conditions change. This flexibility in behavioural expression (often called behavioural plasticity in behavioural ecology, Fig. 1b) is particularly characteristic of the primate order^49^. For instance, maternal style was found to be altered when new individuals were added to the social group of vervet monkeys (*Cercopithecus aethiops*)^50^. In addition to this component of within-individual variation, individuals may differ in the strength of such responses to environmental changes, that is, in the expression of behavioural plasticity (i.e. individual plasticity, or between-individual variation in the adjustment to environmental conditions, Fig. 1c). For instance, some individuals may adjust their behaviour to each environment they encounter, while others may not^51,52^. Although they have largely been studied separately, behavioural type (the average behavioural expression of an individual) and individual plasticity tend to be linked, and flexibility can only be fully understood when the individual level of variation is explicitly addressed. Dingemanse et al. (2010)^51^ argue that studying these two phenomena together is essential to accurately interpret individual variation and to gain the most comprehensive insight into variability. Such an approach, which is usually specific to behavioural ecology, has already been applied to social and spatial behaviour in several species. For example Hertel et al. (2020)^53^ used Generalized Linear Mixed Models to assess reversible and intrinsic individual variation in the spatial movements of African elephants, and Revathe et al. (2025)^54^ used a similar method to investigate individual differences and plasticity in the maternal behaviour of orangutans. Very little is known about between-individual variation in behavioural type and individual plasticity in primate communicative behaviour^38^ but a recent multi-level study (including individuals of two different species in two different research settings) using this method on the infant-directed communication of orangutan mothers provided the first evidence for individual plasticity in primate communication^38^. This proof-of-concept study showed significant differences in the modification of mothers’ behaviour across contexts, with some showing greater responsiveness to their infants than others, suggesting that the degree of behavioural plasticity varies among individuals and paving the way for more investigation of this type.

Here, we aim to expand upon this preliminary work by examining individual variation and plasticity in the communicative behaviour of chimpanzees in two different research settings. Chimpanzees, our closest living relatives along with bonobos, have been extensively studied for their communicative repertoire across modalities (e.g.^13,37,42,55,56)^, providing a solid basis for more in-depth research on the communicative strategies of great apes. Vocal, gestural and facial signals all play major roles in chimpanzee communication, but they are often not used for the same social goals, and particular signals can be more or less tightly linked to specific outcomes. This has been identified in gestural expression: the gesture “leaf-clip” is used only to acquire sexual attention, while “grab” and “pull” are used toward two or more outcomes^56^. The multimodal use of facial signals and vocalizations can also be expressed flexibly by chimpanzees, with facial signals not necessarily being paired with their typically associated vocalisations, which can also lead to altered meaning^57,^ and ten different context-specific vocalizations being expressed in the wild^58^. Furthermore, chimpanzees adapt their communicative strategies to the attentional state of the recipient: silent gestures are preferably chosen when the signaller is in the recipient’s visual field, whereas physical contact is preferred when the signaller is out of sight^14^. This directed, purposeful use of gestures reflects their intentional nature^14^as well as their behavioural plasticity.

The principal goal of this study was to examine the sources of variation in the communicative behaviour of adult zoo- and sanctuary-housed chimpanzees living in multiple social groups through a multimodal approach. To evaluate both the production and perception of multimodal communication, this study distinguished two different facets of it, building on Fröhlich et al. ^11^. *Multicomponent (MC*) communicative acts involve multiple signals (of the same or different signal types, i.e. vocal, bodily, manual, facial components) produced simultaneously by the sender. *Multisensory (MS)* communicative acts are those that are received and processed through several sensory channels (e.g. auditory, visual, tactile). Both communicative strategies might be expressed in the same communicative act (e.g. the combination “touch other + bared-teeth display” is multicomponent with the combination of a limb gesture and a facial signal and multisensory with the combination of the tactile and visual sensory channels) or not (e.g. a “big loud scratch” only includes one gestural component but involves both the visual and auditory sensory channels, while “bipedal stance + reach” involves a body and a limb signal but only the visual sensory channel) and are categorised independently for each communicative act.

This study had four core objectives. The first aim was to explore the specific types of MC and MS acts used by the study subjects in order to better visualise which specific combinations of signals and sensory channels are expressed and at which rates, and to add precision to our current knowledge of the role of multimodality in the communicative system of chimpanzees (but see^37,40,41,59^).

Second, we examined the extent to which individual (e.g. sex, age) and environmental (e.g. research setting, social group) factors influence both MC and MS signal use. We hypothesized that socio-demographic parameters, especially sex and research setting, determine whether these two communicative strategies are expressed. We predicted that sanctuary-housed and female individuals likely combined signals and sensory channels more often than their zoo-housed and male counterparts. Indeed, the more restricted space available in the zoos compared to sanctuaries induces higher levels of proximity. As a consequence, zoo-housed chimpanzees experience reduced social uncertainty and have less need to resort to complex communicative strategies^60^. Similarly, females were expected to use more MC and MS acts than males, reflecting the differential sociality level and social challenges between the sexes^61,62^. Chimpanzee females are typically lower ranking than males, and might, as a consequence, more often face unpredictable interactions in which they would benefit from communicating unambiguously and conspicuously. Individuals from different social groups might also express variation in their communicative signal use, as differences in size and composition of groups (e.g. sex ratio, presence and availability of juveniles) might impose different social constraints on their members (e.g. affiliation rates^63^ or feeding tolerance^64)^.

Third, we investigated whether individual chimpanzees differed in the expression of these strategies on average, regardless of possible socio-demographic differences and other confounding factors (e.g. sex, social context), given that consistent and repeatable differences in social behaviour over time have already been identified in both captive and wild chimpanzees^45,65^. These individual differences in sociality are likely reflected in the chimpanzees’ communicative behaviour as well, we thus expected individuals to consistently express varying levels of MC and MS acts, irrespective of individual (sex, age) and environmental (social context, research setting) parameters.

Finally, we explored the possibility that chimpanzees might shift their communicative strategies between behavioural contexts, and also differ individually in how they do so, using the behavioural reaction norm framework introduced above. In chimpanzees, a single signal can be used to meet different purposes and different signals can lead to the same outcome at the *population level*^17,66,67. Thus, ^it can be assumed that *individuals* flexibly adjust their communicative strategy to the context in which the signaller was involved prior to the initiation of the interaction (i.e. social vs. non-social contexts). Specifically, some individuals may combine signals more often if they are not already engaged in a social activity prior to signalling, e.g. due to different prior interactional experiences. Alternatively, it is also possible that we do not find any evidence for individual plasticity in the studied contexts, as shifts of communicative behaviour in the same direction may be highly adaptive across all individuals in a certain group or setting. In this case, we would expect socio-environmental (e.g. setting) or demographic (e.g. sex) effects to override individual-level effects.

Our results showed that the signaller’s research setting and sex, but not social group, affected the multimodal use of the communicative repertoire, but also that chimpanzees expressed consistent differences in behavioural type for both MC and MS communicative strategies. Moreover, plasticity in relation to the behavioural context was identified for both communicative strategies, but individuals differed in their expressed plasticity only in their use of MS acts. These findings demonstrate the importance of considering individual-level variation in the investigation of great ape communicative behaviour.

## Materials and methods

### Data collection

Data on zoo-housed chimpanzees were collected in two social groups at Zoo Leipzig, Germany, from January to March 2022 (at that time, group A comprised 21 individuals, including 13 adults; group B comprised 6 adults) and one social group at Leintalzoo from May to June and from September to November 2022 (group L, 33 individuals, including 30 adults). In Leipzig, individuals from both groups were housed in indoor enclosures with natural dirt ground but no vegetation and food enrichment boxes. Enclosure A (ca. 200 m^2^) contained a vertical wooden structure with resting platforms while enclosure B (ca. 50 m^2^) was constructed on two levels connected by a stair-like structure. In Leintalzoo, chimpanzees are housed in an enclosure containing an indoor (329.82 m^2^concrete ground) and an outdoor (958.25 m^2^ natural dirt ground) section available freely throughout the day, divided into several connected areas (both horizontally and vertically) and containing bedding and enrichment (logs, ropes, etc.) structures but no vegetation^68^. Data on sanctuary-housed chimpanzees were collected at Chimfunshi Wildlife Sanctuary, Zambia, between July and August 2022 from two social groups: group C2 with 56 individuals, including 29 adults; and group C4 with 13 individuals, including 9 adults. The two sanctuary groups were kept in fenced enclosures of 65 ha and 25 ha, respectively. Each enclosure contained native fruit groves, grasslands, and densely forested miombo woodland, as well as an indoor area for feeding and medical check-ups^69^. Only individuals over the age of 15 years were included in this study to ensure the examination of mature communicative repertoires^45^. However, subjects under 15 who exhibited adult-like behaviours, such as actively participating in reciprocated grooming with adults and adolescent females with infants, were also included (one additional 13-year-old in group L, two 14-year-olds in group C2, and one 13-year-old in group C4; see electronic supplementary material, Tabs. S1 and S2 for detailed information on subjects and group composition).

Dyadic social interactions were recorded using a high-definition camera (Panasonic HC-VXF 999) and an external directional microphone (Sennheiser MKE 600) following a 15-minute focal sampling scheme, which was complemented by *ad libitum* sampling at Chimfunshi, due to the subjects’ unpredictable availability for observation in the sanctuary enclosures. Within the observational follows, only the social interactions (of all types, defined as behaviours involving at least two individuals) of the focal subject, both as signaller and recipient of communicative acts (defined as the smallest communicative unit of either one signal expression, or multiple signals occurring simultaneously, i.e. MC act), and with all conspecifics (adults and juveniles) were recorded. At the zoo, data collection was carried out during opening hours (from 9 am to 5 pm), while it happened mainly around feeding sessions (twice a day, 11:30 am to 1:30 pm and 1:30 pm to 3:30 pm) and availability for observation in the sanctuary groups. The enclosures at Chimfunshi were very large (see above) and chimpanzees roam freely in their dedicated area, which prevented systematic focal follows of the individuals. Therefore, data were collected on most days (five to seven days a week, from 8 am to 4 pm) when the sanctuary-housed chimpanzees were relatively close to the fences and the feeding area and visible. The observation order of all individuals was randomized and ca. 900 h (ca. 660 h for the zoo setting and 200 h for the sanctuary setting) of focal observations were conducted (see electronic supplementary material, Tab. S2, for detailed information on sample size per individual).

Ethical approval for the study on captive chimpanzees was granted by the Chimfunshi Research Advisory Board (CRAB) as well as the research boards of the Zoo Leipzig and the Leintalzoo Schwaigern.

### Video coding procedure

A total of 6925 (zoo: 3013, sanctuary: 3912) video-recorded intraspecific communicative acts were coded in BORIS v 7.13^70^ using a list of 96 communicative signals based on those reported in Hobaiter and Byrne^14^ and Wilke et al.^37^ including vocalizations, manual and body gestures, and facial signals. This study included communicative behaviours that were mechanically ineffective and emitted in a dyadic interaction by a signaller, presumably to elicit a behavioural response by the receiver^56^. This definition thus excluded mechanically effective acts involving physical force (that is, when the “social goal” is achieved without a voluntary action from the recipient). Since this study focused on dyadic interactions, signals that were presumably directed towards several recipients, such as most of the pant hoot vocalizations, or signals produced in ambiguous interactions (e.g. multi-party conflicts), were excluded from the dataset. Additionally, ambiguity was reduced by focusing on signals expressed to initiate an interaction or elicit a behavioural change from the recipient. Signals emitted to maintain a joint behaviour (e.g. during play) were not considered, as they do not fit this definition. Furthermore, even though the range of signallers was limited to mature individuals, no restriction was placed on the recipients of the signals which therefore included both other mature and immature individuals.

In addition to the identities of the signaller and recipient, several descriptive modifiers were coded for each signal instance, based on a previous study on orangutan communication^11^: behavioural context of the signaller (i.e. whether the signaller was engaged in a social or a non-social activity, with the recipient of the signal or other individual(s), prior to the emission of the signal; see electronic supplementary material, Fig. S1 and Tab. S3 for detailed information on the coded contexts), the sensory modalities through which the signal was likely perceived (i.e. auditory, visual, tactile or seismic), the combinatory use of the signal (whether or not it was simultaneously associated with another signal and if so of which type – namely bodily, manual, facial or vocal), the recipient’s attentional state, and the social goal of the signal. Eighteen social goals were identified, based on Hobaiter and Byrne (2014): (1) acquire object, (2) attend to specific location, (3) climb on me, (4) dominance, (5) affiliation/greeting, (6) move away, (7) mover closer, (8) sexual attention, (9) reposition body, (10) seek reassurance, (11) give reassurance, (12) start grooming, (13) start play, (14) stop action, (15) travel with me, (16) tolerance, (17) follow me – sex, (18) other^56^ (see electronic supplementary material, Tab. S3 for definitions of the coding scheme and the modifiers).

Inter-rater reliability was assessed using Cohen’s Kappa coefficient (K^71^), to ensure that coding was consistent between all three coders, which was the case (signal: K = 0.68; behavioural context: K = 0.80; signal combination: K = 0.87; sensory channel: K = 0.87; recipient attention state: K = 0.86; social goal: K = 0.85; 50 videos – including 142 signal instances – were used for the zoo groups and 48 videos – including 130 signal instances – for the sanctuary groups).

### Statistics and reproducibility

After excluding incomplete entries, the total dataset consisted of 6865 communicative acts available for descriptive analyses on multimodality and regression analyses on between- and within-individual differences in multimodal strategies.

In line with previous work on signal complexity in great apes^11,52^ two communicative strategies were analysed, coded as binary variables: multicomponent acts (MC), which represent the instances where at least two signals co-occurred in the same communicative act, regardless of their behavioural type (gestural, facial or vocal; fixed combinations of signals of different communicative domains that necessarily co-occur, such as scream and scream face, were not included as a signal combination), and multisensory acts (MS), namely the perception of signals through at least two sensory channels (i.e. auditory, tactile, visual, seismic) within the same communicative act.

The behavioural reaction norm (BRN) framework used in behavioural ecology^51,52^ was used to partition variation in communicative strategies into its between- and within-individual sources. This method allows assessing the interaction between behavioural types and behavioural plasticity by plotting them along an “environmental” scale (e.g. behavioural context): the intercept mirrors the individual’s behavioural type and the slope its behavioural plasticity. If the slope equals zero, the individual does not express context-driven variation in its behavioural response^38,51–53^. When multiple measures of the same individuals across contexts are available, a random regression analysis can be used to quantify (i) between-individual variation in behavioural type, (ii) behavioural plasticity (i.e. the adjustment to environmental conditions), as well as (iii) the correlation between the two across individuals, (i.e. between-individual variation in behavioural plasticity).

First, the different types of MC and MS communicative acts were investigated in order to determine which specific signal types on the one hand and sensory channels on the other hand were most often combined. Specifically, the proportion of each combination was calculated at the individual level and compared between settings and sexes.

Second, generalized linear mixed models were fitted with a binomial error structure and logit link function, using the R package lme4 (v. 1.1-35.1^72^), to analyse between-individual variation in behavioural type, specifically in the expression of MC and MS communicative acts. These models were used to assess the effects of individual and socio-environmental parameters on the use of these communicative strategies. In all models were included the following fixed effects: signaller’s age (in years, covariate, range = 13–56, z-transformed), signaller’s sex (2 levels: female, male), research setting (2 levels: zoo, sanctuary), the recipient’s attention state (2 levels: attending, not attending), and the behavioural context just before signal production (2 levels: social or non-social, based on whether the signaller was in association with another individual prior to the emission of the signal). Signaller (82 levels), social goal (18 levels, see above) and recipient identity (128 levels) were included as random effects. This allowed the mean behavioural expression (i.e. intercept) to vary among signallers, social goals and recipients. Repeatability was calculated for the two response variables (MC and MS) using the R package rptR (default settings, v. 0.9.22;^73^) to estimate the amount of variation in the response variable explained by random effects (typically, signaller identity) or other grouping factors in the mixed models^74^. Social group was initially included as another random effect, but it was removed because it did not explain any variance (repeatability test for MC: *R* = 0, s.e. = 0.001, 95% confidence interval [CI] = 0–0.005, *p* = 0.5; MS: *R* = 0, s.e. = 0.001, 95% CI = 0–0.005, *p* = 1). To ensure the datasets were representative of the chimpanzee’s communicative behaviour, only individuals that contributed more than two datapoints were included.

Third, to test whether individuals differed in how they shifted their communicative strategies across contexts (i.e. behavioural plasticity), models with random intercepts and random slopes were fitted, based on Hertel et al. (2020)^53^ and Fröhlich et al.^38^. Specifically, in addition to the previously described model, another model was fitted, including the same fixed and random effects as described above and a random slope over an “environmental gradient” (here a behavioural context contrast: social vs. non-social contexts). Two models were thus fitted for each communicative strategy. We compared these models to the models without a random slope using a likelihood ratio test (LRT)^75^. The strength of the models was insured by only including individuals that contributed more than two datapoints to each behavioural context.

The absence of collinearity between predictor variables was confirmed using variance inflation factors (VIFs;^76)^ from models including only the fixed effects (max VIF = 1.19). To test whether signaller identity played a statistically significant role, we also compared the full models to a null model without the random intercept and slopes effects using an LRT. All statistical analyses were conducted using R v 4.3.1^77^.

## Results

MC acts comprised 17.3% (*N* = 1186) and MS acts comprised 42.7% (*N* = 2931) of the total number of 6865 coded communicative acts. On average, 15.6% (range 0–45.9%) of individuals’ communicative acts were MC and 39.9% (range 9.4–68.0%) were MS. Overall, most communicative acts were produced when the subjects were involved in non-social contexts (*N* = 4992; 72.7% of the dataset), and most of the communicative acts were expressed by sanctuary individuals (*N* = 3852; 56.1% of the dataset). Tab. 1 provides an overview of how MC and MS acts were broken down by social group and behavioural context.

**Table 1:** Distribution of signal cases across different groups, contexts and communicative strategies.

	Social groups					Context		Total
	Group A	Group B	Group C2	Group C4	Group L	Non-social	Social	
Multicomponent communicative acts								
Non-MC	1250	273	2398	670	1053	3970	1566	5644
MC	226	47	729	46	138	997	160	1186
Multisensory communicative acts								
Non-MS	783	218	1637	476	813	2786	1070	3927
MS	698	102	1488	245	398	2203	659	2931

### Types of expressed signal and sensory combinations

In a first step, the specific types of signal and sensory combinations were explored. Due to the number of categories and the widely contrasting amount of data in each of them, advanced statistical tests could not be run, but a descriptive overview of the types of MC and MS acts observed is presented in this section. Males and females did not seem to differ systematically in the specific types of MC (e.g. manual-vocal) acts used (Fig. S2a) nor did the settings (Fig. S2b).

The proportion of combined signal use varied dramatically between individuals, ranging from 0 to up to 100% for some combinations. Within a single MC communicative act, individuals mostly combined only two signals, while the combination of more than two signals was rarer, with the exception of the relatively common Facial + Manual + Vocal combination (11.1% of the MC acts; on average 12.2% of the individual repertoires). The most frequent combinations were Manual + Vocal (24.7% of the MC acts; on average 17.4% of the individual samples) and Body + Vocal (14.5% of the MC acts; on average 10.9% of the individual samples).

With regard to MS acts, we found that Tactile and Visual sensory channels were most commonly associated (Fig. S3; 97% of the MS acts; on average 95.6% of the individual samples), followed by Auditory and Visual sensory channels (1.3% of the MS acts; on average 1.7% of the individual samples). There appeared to be no major differences between the sexes (Fig. S3a) or the setting (Fig. S3b).

### Effects of individual and environmental variables on the expression of multimodal strategies

Generalized linear mixed effects models (GLMMs) were used to assess the effects of individual and environmental parameters on the two response variables (proportion of MC and MS communicative acts). Age, sex, recipient’s attention state and behavioural context were included as fixed effects and signaller identity, recipient identity and social goal as random effects. For both response variables, setting had a significant effect. The average expression of MC acts was significantly higher in sanctuary-housed individuals compared to zoo-housed individuals (estimate ± s.e.m. = 0.497 ± 0.176, *p* = 0.005), when the subject was involved in social compared to non-social contexts (0.346 ± 0.124, *p* = 0.005) and in females than in males (-0.337 ± 0.162, *p* = 0.037). The average expression of MS acts was also significantly higher in sanctuary-housed individuals compared to zoo-housed individuals (0.293 ± 0.132, *p* = 0.027) and when the recipient was visually attentive (2.815 ± 0.132, *p* < 0.001), but there was only a positive trend when the subject was involved in social compared to non-social contexts (0.189 ± 0.097, *p* = 0.052). Age did not have an effect on any communicative strategy (MC: 0.066 ± 0.073, *p* = 0.369; MS: 0.077 ± 0.057, *p* = 0.179; see electronic supplementary material, Tab. S4, for detailed information on the model outputs).

### Individual variation in behavioural type for the multimodal strategies

To investigate whether individual chimpanzees differed in their communicative strategies on average, the previously introduced GLMMs with random intercepts only were fitted. The full model explained behavioural variation significantly better than the respective null model (which excluded signaller identity as a random effect) for both response variables (MC: χ^2^ = 64.551, *p* < 0.001, *df* = 1; MS: χ^2^ = 72.808, *p* < 0.001, *df* = 1). This means that considering variation between individuals in the models improved their explanatory power.

**Figure 2:**
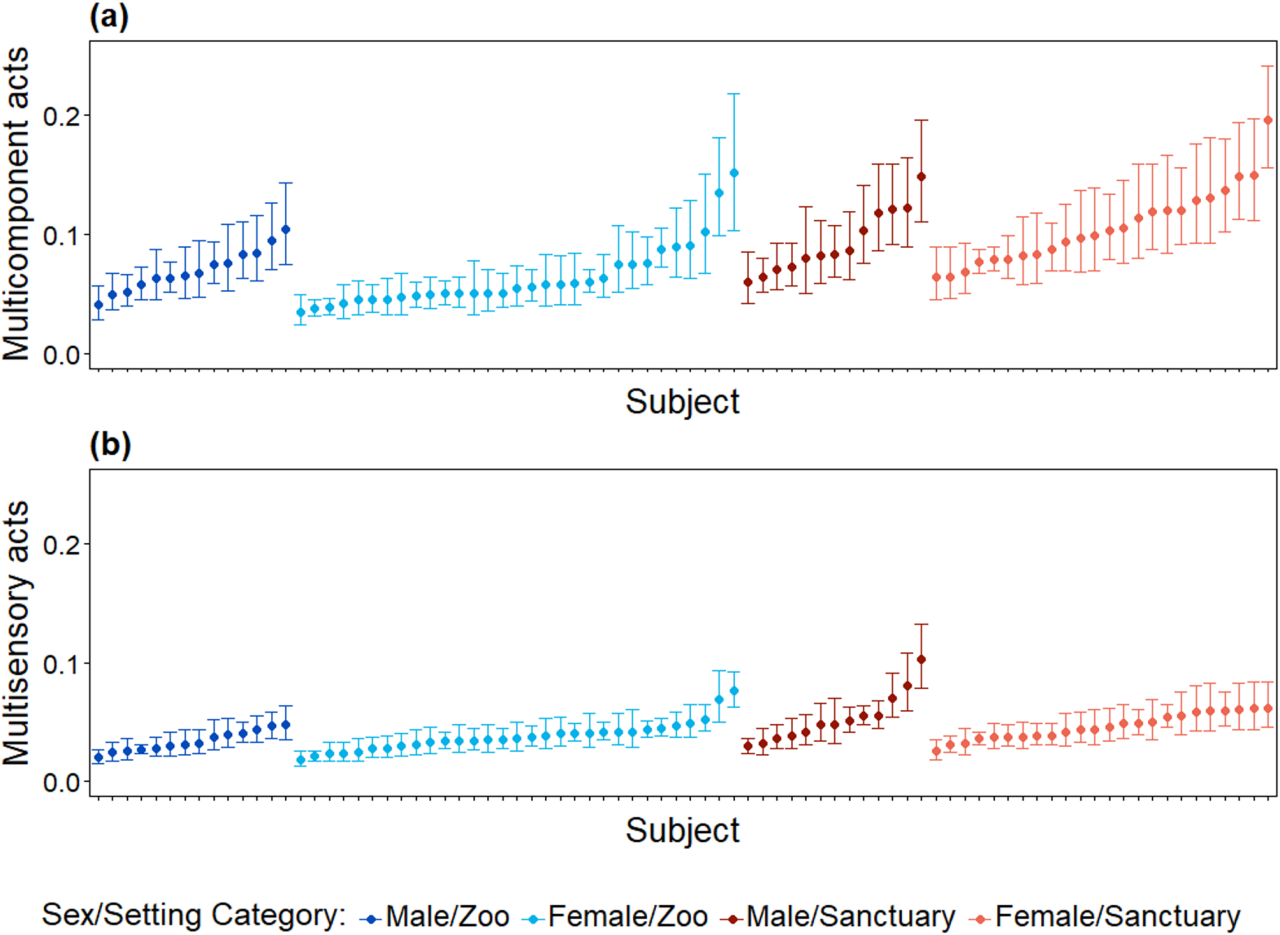
Between-individual variation in multimodal behavioural types in adult chimpanzees. Panels show individual behavioural types for multicomponent (**a**) and multisensory (**b**) communicative acts. Plotted are individual random effect intercepts (best linear unbiased predictors, BLUPs) from models examining variation in the expression of multicomponent and multisensory communicative acts. Colours represent the different sex/setting categories.

For both MC and MS strategies, significant repeatability was found for signaller identity (MC: *R* = 0.025, s.e. = 0.008, 95% confidence interval [CI] = 0.008–0.037, *p* < 0.001; MS: *R* = 0.024, s.e. = 0.006, 95% CI = 0.012–0.035, *p* < 0.001), recipient identity (MC: *R* = 0.023, s.e. = 0.007, 95% CI = 0.006–0.036, *p* < 0.001; MS: *R* = 0.014, s.e. = 0.005, 95% CI = 0.004–0.023, *p* < 0.001) and to a larger extent for the social goal (MC: *R* = 0.156, s.e. = 0.058, 95% CI = 0.038–0.257, *p* < 0.001; MS: *R* = 0.176, s.e. = 0.051, 95% CI = 0.073–0.27, *p* < 0.001). This means that around 2.5% of the remaining variance in MC and MS acts after controlling for confounding effects of age, sex, research setting, and behavioural context could be attributed to differences between individuals. Specifically, some individuals consistently expressed more MC (Fig. 2a) or MS (Fig. 2b) acts compared to others, and these differences were not primarily caused by predictable differences between socio-environmental (research setting or behavioural context) or individual (age, sex) parameters.

### Individual variation in behavioural plasticity for two multimodal strategies

To examine whether chimpanzees differ in how they shift communicative behaviour across social conditions, two models for each response variable (i.e. MC and MS acts) were compared: one with a random intercept for signaller identity (i.e. allowing the response variable to vary among signallers, see above), and one with a random intercept for signaller identity and a random slope for behavioural context within signaller identity (i.e. allowing the slope to vary among signallers).

**Figure 3:**
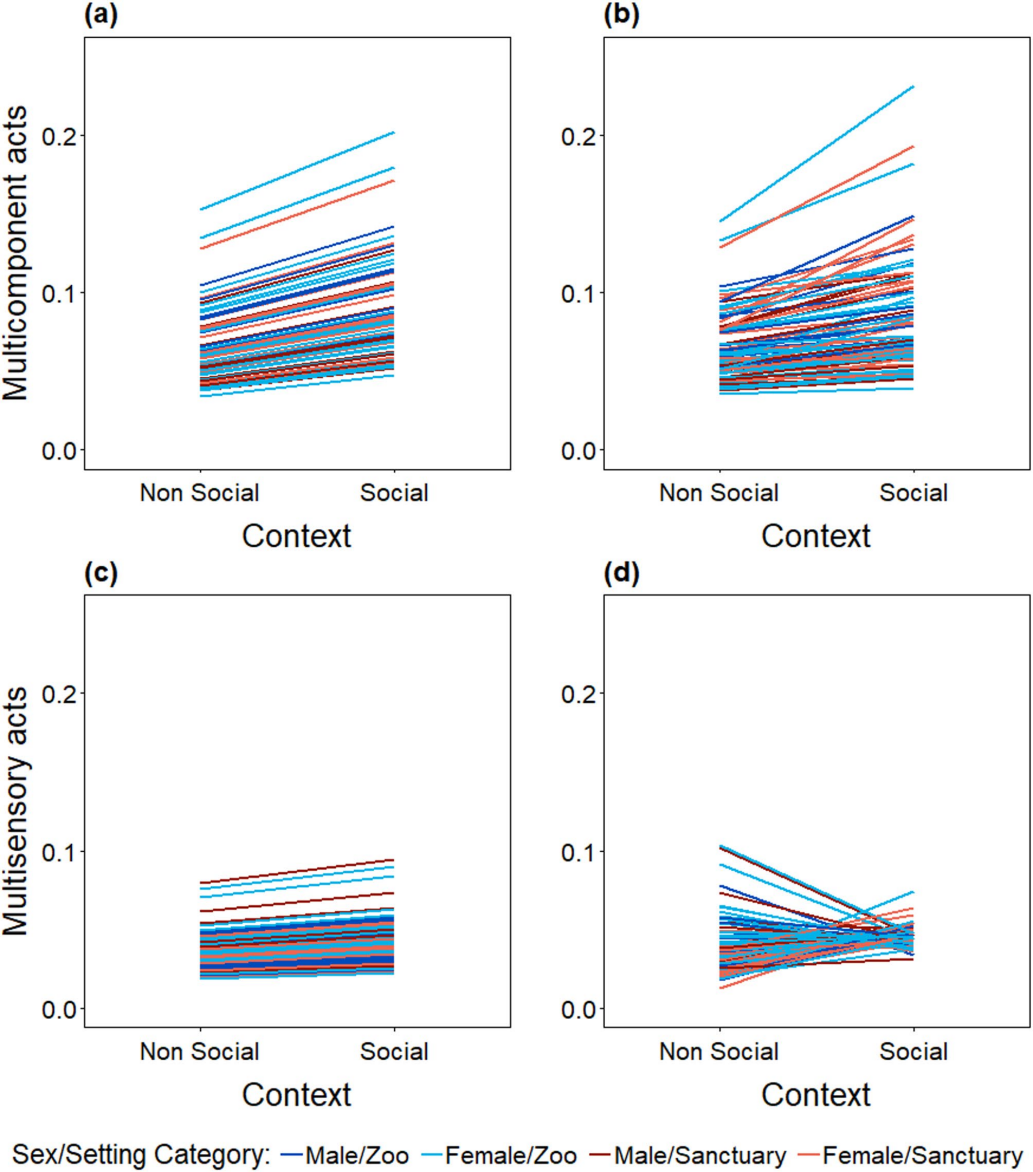
Individual shifts in chimpanzees’ expression of multimodal communication in social versus non-social contexts. Panels show shifts in the use of multicomponent (**a**,**b**) and multisensory (**c**,**d**) communicative acts. (**a**,**c**) show prediction lines assuming individual chimpanzees adjust their behaviour in the same way between behavioural contexts (random intercept). Because predictors were back-transformed, the lines are not strictly parallel as they are on the logit-scale (i.e. only the intercepts vary). (**b**,**d**) show prediction lines assuming individual chimpanzees differ in the extent to which they change their behaviour between behavioural contexts (random intercept and slope). The lack of differences between plots **(a)** and **(b)** shows that the random slope does not add significant explanatory power to the model. Colours represent different sex/setting categories.

As previously mentioned, MC communicative acts were used more often when the individuals were involved in social than in non-social contexts (Tab. S4), but the model including the random slope did not add any explanatory power when compared to the simpler model (χ^2^ = 1.295, *p* = 0.730, *df = 3;* Fig. 3a, b). This means that slopes did not significantly differ between individuals (i.e. they appear parallel on Fig. 3b, see Fig. 1c). Thus, although individuals showed plasticity in their use of MC acts according to the behavioural context, they all did so in the same manner (Fig. 3b). The comparison of the two models showed a better fit of the more complex model (including the random slope for behavioural context within signaller identity) only for the use of MS acts (χ^2^ = 44.5055, *p* < 0.001, *df = 3;* Fig. 3c, d) : the slopes differ among individuals. This indicates that not only do individuals adjust their use of MS communicative acts to the context of the interaction (slope ≠ 0, Fig. 1b), they also do so in different ways (non-parallel slopes, Figs. 1c and 3d). Most of the individuals expressed a higher proportion of MS acts when already engaged in social contexts (Tab. S4), others showed no plastic response, and some even expressed the opposite pattern (Fig. 3d; see electronic supplementary material, Tabs. S4 and S5, for detailed information on the models estimates).

## Discussion

Multimodal approaches in studying primate communication are still severely underrepresented^78^ and the proximate factors impacting complex communicative strategies are still poorly understood. To address this research gap, the goal of this study was to partition variation in multimodal communicative strategies of zoo- and sanctuary-housed chimpanzees into its social, between- and within-individual sources (Fig. 1).

Looking at the role of socio-demographic parameters in the multimodal signalling of chimpanzees, a significant effect of the research setting was found on the expression of both MC and MS communicative acts. Sanctuary-housed individuals combined signals and sensory channels more frequently than the zoo-housed individuals, in line with the prediction. Despite both settings offering limited space and scheduled feeding times, there are also major differences. The studied sanctuary-housed chimpanzees live in far bigger enclosures than the zoo-housed ones, allowing them to engage in more wild-typical behaviour, such as fission-fusion dynamics^79^. In contrast, zoo-living individuals are forced to stay in relatively close proximity to one another. These differences can have far-reaching consequences for the social dynamics within groups. Social interactions come with their load of uncertainty, and thus aggression risk for the interaction partners. As a consequence, individuals may use a variety of strategies to limit ambiguity in order to regulate social relationships with group members^80^ and avoid misunderstandings or communicative failure. The MC and MS use of communicative signals may be highly beneficial as it allows for better detection and interpretation of messages. It might thus be particularly important to invest in such strategies, especially when interactional contexts can change very quickly: wild chimpanzees have been shown to express greater signal complexity in contexts of high social uncertainty^60^ and orangutans to rely more on MC communicative acts in less predictable interaction outcomes and on MS communicative acts to improve effectiveness^11^. Because zoo-housed individuals spend more time in close proximity to all individuals in the group, they are more familiar with each other and probably need to rely less on complex signalling than sanctuary-housed individuals^11,60^. This is consistent with previous research showing that repetitions of vocal greeting signals are rarely used among strongly bonded individuals, but are frequently used among distant dyads^81^.

Another explanation for this might be the differing physical and ecological environment between the two settings: the bigger enclosures of the sanctuary potentially increase the distance between the interaction partners and the richer vegetation in this setting causes visual noise. As a consequence, sanctuary-housed chimpanzees might increase their use of MC and MS acts as a compensation strategy, as has been identified in frogs (e.g. *Staurois parvus*^82^).

Females used significantly more MC acts than males, but not more MS acts, which is partly in line with the predictions. Similarly to setting differences, the strong and cooperative male-male social bonds^61,83,84^ might require less complex communicative strategies than female-female communication to successfully negotiate interactions, although the traditionally ascribed asocial nature of female chimpanzees has been heavily debated (e.g.^85^). Moreover, males and females might signal to conspecifics of the opposite sex with widely different social goals. For example, females may express more submissive, reassurance-seeking signals (e.g. “greetings”) towards males than males do towards females^86^. These types of interactions pose a particular risk for the signallers, who are typically lower-ranking and have less fighting ability than their recipients. The use of MC acts might thus be more important for females than for their male counterparts. Although this was not investigated in detail, the social goal of the interaction appears to play an important role in the use of both MC and MS communicative strategies. It would be interesting to explore this effect further, especially its interactions with other parameters, such as signaller and recipient sexes, as previous research has shown that these factors influence gestural signalling in chimpanzees^87^.

When examining the distribution of the types of signal and sensory channel combinations, considerable variation between individuals in the same research setting appeared. This was somewhat supported by the repeatability results, which showed that 2.5% of the variance was explained by consistent individual differences, regardless of the social or contextual environment. These values are relatively small compared to other repeatability scores found in chimpanzees and other species^45,88^ and even to a similar study in orangutans^38^. Most of the variation seemed to be explained by the social goal of the interaction (accounting for 16 to 18% of the remaining variance), but other factors, such as context or recipient-related variables (as well as interacting factors, as mentioned above; e.g. sex/social goal), could also contribute to these low values. This would not be surprising given that the social goal of the interaction is a major source of flexibility (means-end dissociation;^12,18–20^). Nevertheless, these results demonstrate that there are consistent individual differences in communicative behavioural types.

These findings may have intriguing implications for the study of evolutionary origins of politeness in human communication. Linguistic politeness in humans is thought of as a means to reduce social friction and soften threatening acts through the use of culturally defined communicative signals and cues^8,89^. Specifically, when interaction partners share a stronger social bond, meaning that they interact often and know each other well, they need to put less effort into their communicative interactions (e.g. they need to go through less steps, such as using “try markers”, to construct a common ground and be understood^6)^. This can be understood as a reduced need to rely on complex communicative strategies such as signal and sensory combinations. This phenomenon is mirrored by our findings, including the differentiated use of both these strategies between zoo- and sanctuary-housed individuals. Similar processes thus seem to occur in the communication of human and non-human primates, including the potentially similar reliance on social norms (namely the behavioural expression based on the understanding of the social environment and the anticipation of conspecifics’ behaviour, particularly in the case of non-compliance to these norms) that is characteristic of human social behaviour^90^. This has already been identified in the communicative behaviour of juvenile chimpanzees, who were shown to accentuate their rate of play signals to avoid third party intervention when play bouts become ambiguous^91^.

This was represented in this study by the finding that the behavioural context, namely the activity of the signaller prior to signalling, had a significant impact on the use of MC acts, but only a marginal one on that of MS acts. Surprisingly, individuals relied more on MC and MS acts when they were already engaged in a social activity with a conspecific (e.g. grooming or playing) than when they were not (e.g. resting or feeding). One possible explanation may be that, although the signaller was already socially engaged, their social partner might not necessarily be the intended recipient of the communicative act. Thus, switching interaction partners might require even more ambiguity-reducing strategies than initiating a social interaction from a non-social context, since the signaller needs to manage a more complex social environment. Nonetheless, the findings demonstrate that chimpanzees exhibit plasticity in their communicative behaviour, adjusting their expressions in response to changes in behavioural context.

To assess not only whether there was a setting-level contextual shift in communicative strategy, namely plasticity, but also between-individual variation in plasticity, a behavioural reaction norm framework was applied to the dataset. Contrary to our predictions, individuals varied in plasticity in their expression of MS acts but not MC acts, despite the apparent similarity between the two strategies. In particular, individuals seemed to adjust their use of MC acts to the behavioural context they were in prior to the emission of the signal (population-level behavioural plasticity, see above), but all of them did so in a similar way. Fröhlich et al.[38] found variation in plasticity in orangutan mothers in two very different variables, which seems to suggest that such variation is more broadly present in orangutan communicative behaviour. However, a reason for this could be that this study focused on mother-infant interactions and not on all available interaction types, as was done here. Their results might thus specifically reflect variation in maternal styles, which commonly occurs in primates (e.g^.50,92^). Kin relationships and rank could not be controlled for in the present study due to substantial variation in kinship structure across groups and insufficient data on dominance ranks, respectively. However, both factors, along with an affiliation index, could significantly influence the strategies individuals use in their communicative attempts. For example, lower-ranking chimpanzees tend to become more agitated in the presence of a higher-ranking group member, leading them to produce more vocalizations^93^ and modality combinations have been shown to be affected by dominance relationships and familiarity between individuals^11,94^. Future studies should thus take these into account in order to provide a more representative view of the causes of individual differences in multimodal communicative expression.

Another explanation could be that the need to appropriately adjust one’s use of MC acts across behavioural contexts is so critical that it prevents the expression of differences in plasticity. Indeed, the results presented here show that individuals do express differing behavioural types in both social and non-social contexts; there is simply no variation between individuals in the strength and direction of their behavioural adjustment when these contexts change. This probably means that there is a required amount of MC communicative acts that individuals must express when involved in a social context for the interaction to proceed smoothly. Some social interactions are risky for the signaller, since they might be followed by directed aggression from the recipient if the signaller does not assess the situation properly. As mentioned above, individuals might thus rely on strategies to limit ambiguity, but they might also have to follow strict social norms to improve their chances of success^90,91^ as has been found with the greeting behaviour of this species: vocal sequences adhere to specific rules based on the hierarchical and social relationship between social partners^81^. In this case, individuals seem to rely more on MC acts in social contexts, but they probably also *need* to do so in a specific way to minimize the uncertainty of the interaction.

In other words, if increasing one’s use of MC acts is necessary to avoid communicative failure, individuals who decrease their MC acts expression might be faced with serious consequences (e.g. aggression, exclusion from food resources). In this case, variation in behavioural plasticity among individuals is regulated by the strength of the risk associated with such interaction: the greater the risk, the more standardized the behaviour, and the weaker the differences in individual plasticity^52,95–97^.

In contrast, chimpanzees were found to vary in how they individually shift their use of MS acts between contexts (individual-level behavioural plasticity). This suggests that, contrary to the use of MC acts, the use of MS communicative acts might not be an aspect of chimpanzees’ communicative behaviour that is particularly important for the success of communicative interactions, which results in less constraint on how individuals use them: MS acts might not need to be standardized. If the costs associated with the misuse of MS acts are low, individuals are not under strong selective pressure in their expression of MS acts, leaving room for individual differences in behavioural plasticity. The finding that chimpanzees did not adjust their behaviour to the behavioural context at the population-level supports this: MS acts were used equally in social and non-social contexts overall, but differences lie at the individual level.

Although they seem closely related, both communicative strategies were not similarly impacted by context changes. Mathot et al. (2011) showed that the payoffs of plasticity are not affected in the same way in all behavioural traits by the frequency-dependency phenomena: some payoffs are negative-frequency dependent (e.g. the benefits of being plastic decrease when more individuals are plastic) and others are positive-frequency dependent (e.g. the benefits of being plastic increase with increasing number of plastic individuals^97)^. Similarly, different communicative strategies seem to be affected to a varying degree by social risks. This result is in agreement with the previous finding that MC and MS communicative acts have different functions in orangutans^11^ and shows that these analyses are highly variable-sensitive: plasticity and variation in plasticity might not be found in, or be adaptive for all communicative strategies and it is important to include all variations of these in the study of animal behaviour in order to properly evaluate it.

## Conclusions

One constitutive feature of human language is its flexibility, which allows it to adapt to the context in which interactions occur — a flexibility partly achieved through multimodality. . However, and despite the importance of the comparative approach in studies on language evolution, individual variation, and especially plasticity, have been neglected in studies on non-human communication systems. This study shows that individual chimpanzees vary in their use of MC and MS communicative acts (i.e. individual variation in behavioural type), which are largely shaped by the setting they live in. Chimpanzees also showed some level of behavioural plasticity between behavioural contexts, but individual variation in plasticity was identified in only one of the two communicative strategies.

Multimodality in chimpanzee communicative flexibility appears to play a similar role as it does in human language: communicative complexity is associated both with the familiarity of, and the adjustments of the communicative behaviour (i.e. plasticity) to, the interaction partner. This underscores the relevance of comparative communication studies in understanding the evolutionary origins of language.

## Supplementary Information

Below is the link to the electronic supplementary material.

Supplementary Material 1

## Data Availability

The datasets and R script that support the findings of this study have been deposited on GitHub: https://github.com/angelelombrey/ALombrey-multi-plasti-Data-and-code.git.
