# Supplementary material for: Flexible use of multimodal communicative strategies in adult chimpanzees
Source: Sci Rep. 2025 Sep 9;15:32384. doi: 10.1038/s41598-025-14835-x (PMC12420796; doi:10.1038/s41598-025-14835-x)
Supplement: Supplementary file 1 — Supplementary Material 1 [file 41598_2025_14835_MOESM1_ESM.docx]

**Supplementary Material**

**Flexible use of multimodal communicative strategies in adult chimpanzees (*Pan troglodytes*)**

Angèle Lombrey, Adriana Luna, Nick Dannenmann, Katerina Harvati, Ulrich Knief, Marlen Fröhlich

Table S1: Information on group composition

| **Research setting** | **Social group** | **Number of subjects** | **Number of male subjects** | **Number of female subjects** | **Total group size** |
| --- | --- | --- | --- | --- | --- |
| Captive | A | 13 | 2 | 11 | 21 |
|  | B | 6 | 1 | 5 | 6 |
|  | L | 30 | 13 | 17 | 33 |
| Semi-wild | C2 | 29 | 8 | 21 | 56 |
|  | C4 | 9 | 5 | 4 | 13 |

Table S2: Subject information and sample size per subject represented by the amount of coded signal utterances

| **Subject** | **Group** | **Setting** | **Sex** | **Age** | **Rank** | **Sample size** |
| --- | --- | --- | --- | --- | --- | --- |
| Alex | B | Captive | M | 21 | 1 | 96 |
| Bambari | A | Captive | F | 21 | 5 | 64 |
| Bea | L | Captive | F | 27 | NA | 13 |
| Bobby | C4 | Sanctuary | M | 28 | NA | 38 |
| Carol | C2 | Sanctuary | F | 25 | NA | 94 |
| Coco | C2 | Sanctuary | F | 37 | NA | 30 |
| Commander | C4 | Sanctuary | M | 22 | NA | 146 |
| Corry | A | Captive | F | 45 | 7 | 240 |
| Daisy | C2 | Sanctuary | F | 17 | NA | 93 |
| David | C2 | Sanctuary | M | 20 | NA | 154 |
| Daza | B | Captive | F | 36 | 3 | 92 |
| Debbie | C2 | Sanctuary | F | 20 | NA | 139 |
| Diana | C2 | Sanctuary | F | 31 | NA | 36 |
| Diz | C2 | Sanctuary | F | 14 | NA | 81 |
| Dolly | C2 | Sanctuary | F | 25 | NA | 277 |
| Donald | L | Captive | M | 28 | NA | 53 |
| Dora | C2 | Sanctuary | F | 33 | NA | 139 |
| Dorien | A | Captive | F | 41 | 6 | 50 |
| Doug | C2 | Sanctuary | M | 19 | NA | 92 |
| Espe | L | Captive | F | 31 | NA | 104 |
| Fetz | L | Captive | M | 16 | NA | 53 |
| Fraukje | A | Captive | F | 45 | 9 | 75 |
| Freddy | L | Captive | M | 35 | NA | 20 |
| Frederike | B | Captive | F | 48 | 5 | 26 |
| Frodo | A | Captive | M | 28 | 1 | 146 |
| Garibal | L | Captive | M | 26 | NA | 29 |
| Gartenzwerg | L | Captive | F | 39 | NA | 107 |
| Girlie | L | Captive | F | 18 | NA | 34 |
| Gregor | L | Captive | M | 33 | NA | 81 |
| Heronemo | L | Captive | M | 37 | NA | 12 |
| Hope | B | Captive | F | 31 | 2 | 33 |
| Ignaz | L | Captive | M | 33 | NA | 28 |
| Jack | C4 | Sanctuary | M | 13 | NA | 154 |
| Jacky | L | Captive | F | 52 | NA | 10 |
| Jeudi | B | Captive | F | 56 | 4 | 39 |
| John | C2 | Sanctuary | M | 15 | NA | 93 |
| Judy | C2 | Sanctuary | F | 26 | NA | 103 |
| Kamboo | C4 | Sanctuary | F | 26 | NA | 24 |
| Kathy | C4 | Sanctuary | F | 23 | NA | 73 |
| Kisha | A | Captive | F | 18 | 8 | 85 |
| Lindi | L | Captive | F | 26 | NA | 22 |
| Little Jane | C2 | Sanctuary | F | 37 | NA | 232 |
| Little Jenkins | C4 | Sanctuary | F | 15 | NA | 31 |
| Lutz | L | Captive | M | 18 | NA | 29 |
| Maggie | C2 | Sanctuary | F | 36 | NA | 62 |
| Maja | A | Captive | F | 35 | 10 | 212 |
| Malia | L | Captive | F | 16 | NA | 52 |
| Mary | C4 | Sanctuary | F | 16 | NA | 20 |
| Masya | C2 | Sanctuary | F | 31 | NA | 57 |
| MaxC | C2 | Sanctuary | M | 15 | NA | 49 |
| MaxL | L | Captive | M | 26 | NA | 52 |
| Mickey | C2 | Sanctuary | M | 34 | NA | 111 |
| Misha | C2 | Sanctuary | F | 34 | NA | 102 |
| Moyo | C2 | Sanctuary | M | 14 | NA | 95 |
| Nana | L | Captive | F | 26 | NA | 56 |
| Natascha | A | Captive | F | 41 | 5 | 59 |
| Negine | L | Captive | F | 22 | NA | 16 |
| Nikki | C2 | Sanctuary | F | 24 | NA | 74 |
| Nina | C2 | Sanctuary | F | 19 | NA | 68 |
| Noel | C2 | Sanctuary | F | 45 | NA | 84 |
| Pan | C2 | Sanctuary | M | 33 | NA | 64 |
| Panya | L | Captive | F | 16 | NA | 18 |
| Para | L | Captive | F | 24 | NA | 49 |
| Paul | L | Captive | M | 31 | NA | 30 |
| Paulinchen | L | Captive | F | 34 | NA | 38 |
| Pippa | C2 | Sanctuary | F | 33 | NA | 113 |
| Pitch | L | Captive | F | 26 | NA | 20 |
| Riet | A | Captive | F | 44 | 3 | 42 |
| Robert | A | Captive | M | 46 | 2 | 25 |
| Sandra | A | Captive | F | 28 | 5 | 114 |
| Scholzi | L | Captive | F | 35 | NA | 39 |
| Scholzon | L | Captive | M | 23 | NA | 30 |
| Sinkie | C4 | Sanctuary | M | 28 | NA | 41 |
| Swela | A | Captive | F | 26 | 7 | 228 |
| Tai | A | Captive | F | 19 | 4 | 142 |
| Tess | C2 | Sanctuary | F | 23 | NA | 224 |
| Tilly | C2 | Sanctuary | F | 21 | NA | 182 |
| Toto | L | Captive | M | 42 | NA | 32 |
| Trixie | C2 | Sanctuary | F | 32 | NA | 75 |
| Val | C4 | Sanctuary | M | 22 | NA | 195 |
| Violet | C2 | Sanctuary | F | 31 | NA | 189 |
| Vis | C2 | Sanctuary | M | 18 | NA | 60 |
| Wazlaw | L | Captive | M | 31 | NA | 40 |
| Zicklein | L | Captive | F | 23 | NA | 39 |
| Zina | L | Captive | F | 18 | NA | 35 |
| Zira | B | Captive | F | 24 | 3 | 34 |
| Zora | L | Captive | F | 13 | NA | 70 |


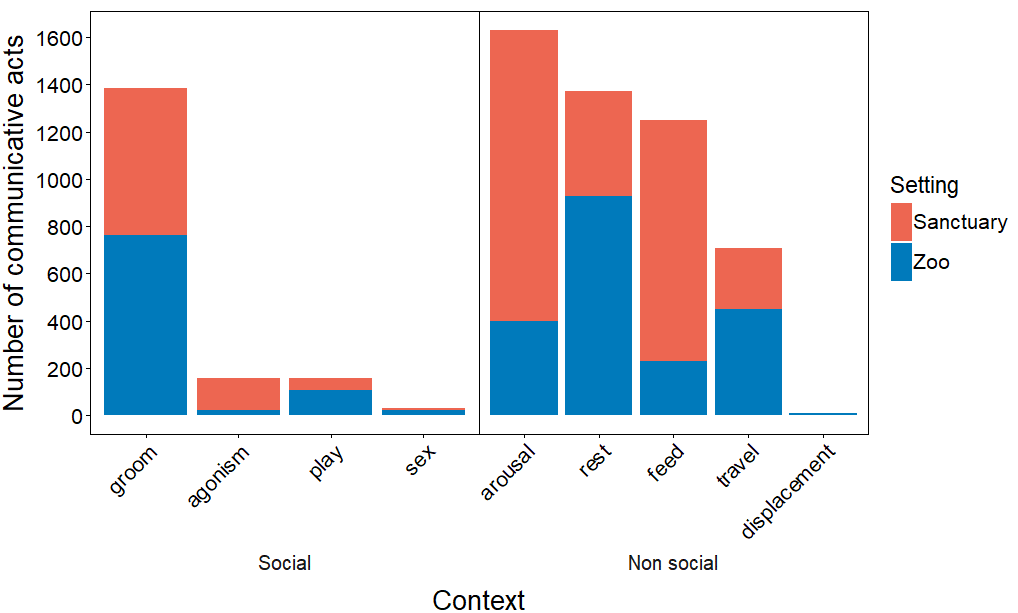


*Figure S1: Distribution of the communicative acts across the nine social contexts recorded. Bars represent the number of communicative acts recorded in each research setting.*

Table S3: Definition of the modifiers used in the BORIS coding scheme (S: signaller; R: recipient)

| **Modifier** | **Definition** |
| --- | --- |
| **Social context** | Behaviour of S just before the emission of the signal |
| agonism | S is engaged in a conflict with either R or other individuals |
| arousal | S is excited and/or stressed out, often showed by loud vocalizations and/or emotional running |
| displacement | S expresses stereotypical behaviours |
| feed | S is eating or being fed |
| groom | S is grooming or being groomed by R or another individual |
| play | S is engaged in a play session |
| sex | S is having sex |
| travel | S is travelling over a long distance |
| rest | S is steady or self-grooming |
| other |  |
| NA | unknown due to poor visibility |
| **Sensory channel** | Sensory channel through which the signal is perceived by R (multiple choice) |
| Auditory | S's behaviour produces a sound and is perceived through hearing |
| Visual | S's behaviour is perceived via eye sight |
| Seismic | S's behaviour is perceived through substrate movement |
| Tactile | S's behaviour is perceived through body contact |
| **Co-occurring signal types** | Combination with another signal type (multiple choice) |
| no | no |
| yes - plus facial | combination with facial expression |
| yes - plus facial fixed | obligatory combination with facial expression |
| yes - plus manual | combination with manual gesture |
| yes - plus body | combination with bodily, non-manual gesture |
| yes - plus vocal | combination with vocalization |
| yes - plus vocal fixed | obligatory combination with vocalization |
| NA | unknown due to poor visibility |
| **Recipient’s attention state** | Visual orientation of R during S's behaviour |
| 0 | S is not in the R’s visual field, that is the R would have to make a major turn with his/her head or body to see the S – R can not see the signal |
| 1 | S is in the visual field or R (the R can see the S without moving their head) or in its periphery (R can see the S from the corner of his/her eye) – R can the the signal |
| NA | Not clearly visible, unknown |
| **Social goal** | Apparent aim of S, as evident by cease of communication and previous behaviour |
| Acquire object | R gives signaller object, e.g. food item |
| Attend to spec location | R adjusts behaviour to focus attention on indicated location |
| Climb on me | R (infant) climbs on signaller’s body |
| Dominance | R acknowledges that S is higher ranking |
| Affiliation/greeting | R reacts in an apparently affiliative way |
| Follow me - sex | R follows S, usually in consortship |
| Move away | R moves away from signaller |
| Move closer | R moves closer to signaller |
| Sexual attention | R responds sexually |
| Seek reassurance | S want R to reassure them |
| Give reassurance | S want R to calm down |
| Reposition body | R moves (and hold) body into indicated position |
| Start grooming | S wants R to groom them |
| Start play | play between signaller and recipient |
| Stop action | R either ceases current behaviour or change behaviour to direct it toward another individual |
| Travel with me | R travels together with S |
| Tolerance | S signals in a submissive way, no particular behavioural outcome is looked for |
| Other |  |
| NA | unknown |
| **Response** | Response of R in reaction to S's behaviour |
| none | No response or ignorance by R |
| Social goal | R responds in a way that seems appropriate to S's signal |
| responds with visual attention only | R looks at S (but no other response) |
| behavioural response - signal | R responds with a signal - "negotiation”; ≠ from a signal-like response produced as an automatic reaction to S's signal |
| behavioural response - move away | R moves away from S |
| behavioural response - aggressive | R reacts in an aggressive or agonistic way to S's signal |
| NA | Not clearly visible, unknown |
| **Recipient** | Recipient ID  Single choice: only dyadic communicative acts are considered - broad behaviour (e.g. vocalizations) for which one precise recipient cannot be identified are not included |


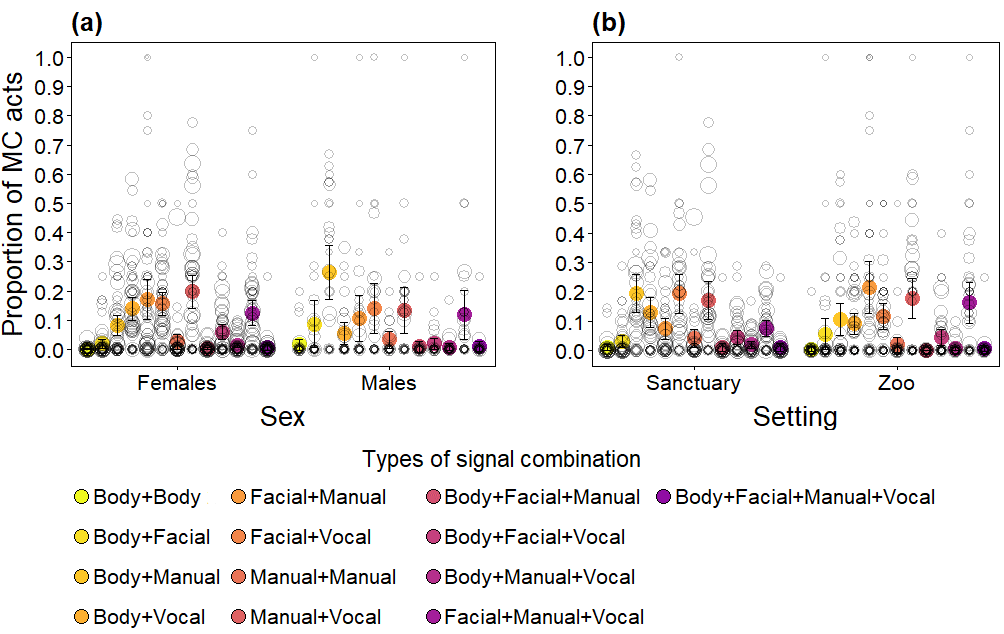


Figure S2: Mean proportions of multicomponent (MC) use for each signal combination. Panel (a) shows MC use across sexes and panel (b) across research settings. Indicated are individual means per MC type (circles) and population means (filled circles) with standard error (vertical lines). Colours represent the types of signal combination and circle size represents the sampling effort per individual (i.e. total number of MC acts recorded per individual).


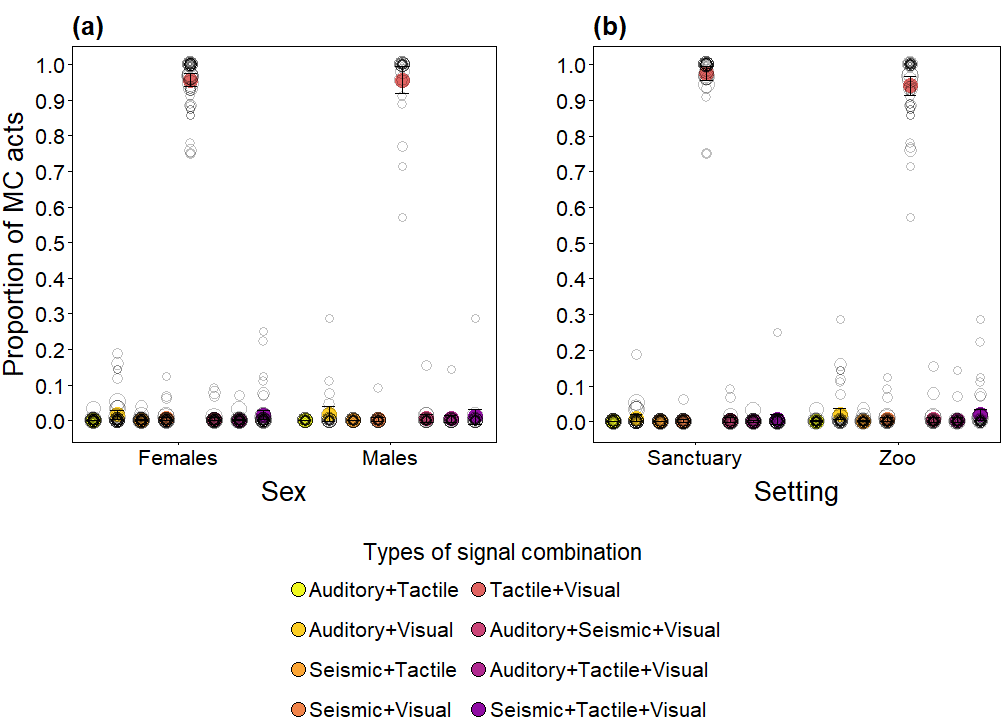


Figure S3: Individual proportion of multisensory (MS) use for each sensory combination. Panel (a) represents MS use across sexes and panel (b) across settings. Indicated are individual means per MC type (circles) and population means (horizontal lines) with standard error (vertical lines). Colours represent the types of sensory channel combination and circle size represents the sampling effort per individual (namely the total number of MC acts recorded for each individual).

Table S4: Fixed effects estimates from the random intercept models (including Subject ID as a random effect) for both communicative strategies

| Fixed effects | Estimate | Std. Error | Z value | Pr |
| --- | --- | --- | --- | --- |
| **Multicomponent acts** | | | | |
| (Intercept) | - 2.721 | 0.346 | - 7.860 | 3.84e-15 *** |
| Age | 0.066 | 0.073 | 0.899 | 0.369 |
| SexM | - 0.337 | 0.162 | - 2.084 | 0.037 * |
| SettingSanctuary | 0.497 | 0.176 | 2.824 | 0.005 *** |
| ContextSocial | 0.346 | 0.124 | 2.781 | 0.005 *** |
| RecAttentionState_YNT | 0.149 | 0.134 | 1.115 | 0.265 |
| **Multisensory acts** | | | | |
| (Intercept) | - 3.341 | 0.309 | - 10. 814 | <2e-16 *** |
| Age | 0.077 | 0.057 | 1.345 | 0.179 |
| SexM | - 0.080 | 0.126 | - 0.632 | 0.527 |
| SettingSanctuary | 0.293 | 0.132 | 2.219 | 0.027 * |
| ContextSocial | 0.189 | 0.097 | 1.942 | 0.052 |
| RecAttentionState_YNT | 2.815 | 0.132 | 21.296 | <2e-16 *** |

Table S5: BRN model comparison estimates for both communicative strategies (ri = random intercept model; ris = random intercept and slope model)

| Model | npar | AIC | BIC | logLik | deviance | Chisq | Df | Pr |
| --- | --- | --- | --- | --- | --- | --- | --- | --- |
| **Multicomponent acts** | | | | | | | | |
| ri | 9 | 5041.326 | 5102.332 | -2511.663 | 5023.326 | NA | NA | NA |
| ris | 12 | 5046.031 | 5127.372 | -2511.015 | 5022.031 | 1.295 | 3 | 0.730 |
| **Multisensory acts** | | | | | | | | |
| ri | 9 | 7437.807 | 7498.847 | -3709.904 | 7419.807 | NA | NA | NA |
| ris | 12 | 7399.303 | 7480.689 | -3687.651 | 7375.303 | 44.505 | 3 | 1.179e-09 |
